# Clinical practice style is associated with greater isotretinoin use for acne in an integrated health system: An ecological study

**DOI:** 10.1016/j.jdin.2025.10.014

**Published:** 2025-11-03

**Authors:** Veena Vanchinathan, Michael Hartmann, Noah M. Contreras, Andrew L. Avins, Amara A. Lieberman

**Affiliations:** a Department of Dermatology, Northern California Kaiser Permanente, San Jose, California; b Division of Research, Northern California Kaiser Permanente; Oakland, California; c Kaiser Permanente Bernard J. Tyson School of Medicine; Pasadena, California; d Department of Dermatology, Northern California Kaiser Permanente, San Jose, California

**Keywords:** acne, database study, isotretinoin, practice, prescription drug managementretinoids

*To the Editor:* Isotretinoin is effective for severe acne^1^ but can be cumbersome for prescribers and patients because of safety and regulatory burdens.^2^ Prior studies show wide variation in isotretinoin prescribing, but few studied real-world data to understand variation in and predictors of isotretinoin use to help reduce treatment barriers and promote wider use for appropriate patients.

We conducted a cross-sectional ecological study within Kaiser Permanente Northern California (KPNC), an integrated system serving 4.5 million members^3^^,^^4^; within KPNC, providers set their own treatment plans without restrictions. Patients aged 13-40 years with ≥2 visits with acne diagnoses from 2016 to 2020 were included. Because we lacked structured data on patient-level acne characteristics, we used an ecological design, akin to “small-area variation” studies of health care utilization.^5^ Patient-level data were aggregated at the level of 15 independent, clinically similar medical-center geographic clusters (“service areas”) for analysis; we examined 20 candidate predictors at the service-area level (Supplementary Appendix, available via Mendeley at https://data.mendeley.com/datasets/t5zswmwyyt/1). The primary outcome was the percentage of patients in each service area receiving ≥1 isotretinoin prescription. We conducted multivariable mixed-effects linear regression to account for service-area clustering; model fit and normality assumptions were tested during each iteration to assess possible overfitting. The model-output beta coefficients can be interpreted as the percent change in a service area’s percentage of patients prescribed isotretinoin associated with a 1-unit change in the predictor variable.

We identified 85,359 KPNC patients enrolled for ≥1 year before and after their entry into the cohort with no isotretinoin prescription prior to 2016. The overall sample was young (median age = 19 years) with a female predominance (68%; Table I); 9639 patients (11.6%) received ≥1 isotretinoin prescription. The numbers of study-cohort patients in each service area ranged from 2492 to 12,078.

**Table I:** Demographic and clinical characteristics of study cohort

Characteristic	All patients (*N* = 83,359)	Never prescribed isotretinoin (*N* = 75,720)	Prescribed isotretinoin at least once (*N* = 9639)
Patient age, y (*n* [%])			
13-17	36,495 (43%)	834 (41%)	5665 (59%)
18-30	33,683 (39%)	30,499 (40%)	3184 (33%)
31-40	15,181 (18%)	14,391 (19%)	790 (8%)
Patient sex (*n* [%])			
Female	58,273 (68%)	53,311 (70%)	4962 (51%)
Male	27,074 (32%)	22,398 (30%)	4676 (49%)
Unknown/other	12 (<0.1%)	11 (<0.1%)	1 (<0.1%)
Race/ethnicity (*n* [%])			
African-American	5032 (5.9%)	4733 (6.3%)	299 (3.1%)
Asian	19,323 (23%)	17,667 (23%)	1656 (17%)
Hispanic	15,703 (18%)	13,850 (18%)	1853 (19%)
White	35,057 (41%)	30,379 (40%)	4678 (49%)
Other/unknown	10,244 (12%)	9091 (12%)	1153 (12%)
Median annual household income (*n* [%])			
$0 – ≤$75,000	27,417 (32%)	24,206 (32%)	3211 (33%)
$75,000 – $≤100,000	22,654 (27%)	20,056 (26%)	2598 (27%)
>$100,000	35,288 (41%)	31,458 (42%)	3830 (40%)
Year of index date (*n* [%])			
2016	30,529 (36%)	26,073 (34%)	4456 (46%)
2017	21,685 (25%)	19,315 (26%)	2370 (25%)
2018	15,078 (18%)	13,698 (18%)	1380 (14%)
2019	10,056 (12%)	9209 (12%)	847 (8.8%)
2020	5485 (6.4%)	5049 (6.7%)	436 (4.5%)
2021	1872 (2.2%)	1749 (2.3%)	123 (1.3%)
2022	565 (0.7%)	539 (0.7%)	26 (0.3%)
2023	89 (0.1%)	88 (0.1%)	1 (<0.1%)

Rates of isotretinoin therapy initiation varied from 7.7% in the lowest-prescribing service area to 17.3% in the highest (risk ratio = 2.2, 95% CI: 2.0-2.6; Fig 1). Two predictors were independently and statistically significantly associated with higher isotretinoin prescription rates: service areas with fewer years between the index date and first isotretinoin prescription (beta = −0.07, 95% CI: −0.12 to −0.03, *P* = .006) and service areas with higher percentages of prior oral antibiotic prescriptions (beta = 0.40, 95% CI: 0.16 to 0.68, *P* = .004). A positive but nonsignificant association was observed between the proportion of advanced practice practitioner (APP)-initiated initial isotretinoin prescriptions and overall prescribing rates (β = 0.36; 95% CI, −0.01 to 0.72; *P* = .0526). All associations were independent of other service area-based estimates of patient age, race, gender, patient-provider gender concordance, median household income, and language-interpreter usage, which were not significant predictors. All KPNC APPs have similar qualifications and licensure; service areas differ in APP numbers and clinical assignments.

**Fig 1:**
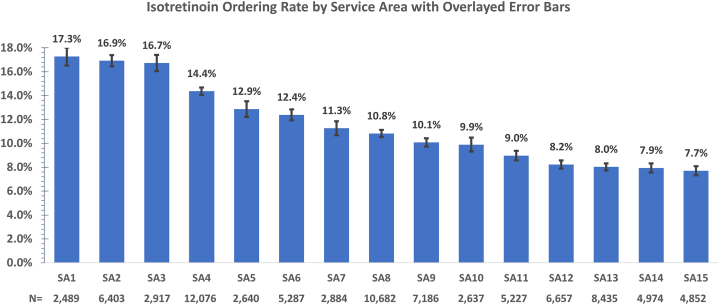
Acne vulgaris. Percentages of acne patients for whom at least 1 isotretinoin prescription was provided in each service area. Percentages are depicted per medical-system service area (generally including 1 to 3 medical centers). N’s represent denominators (number of patients with acne diagnoses) per service area. Error bars represent 95% CIs.

Limitations include a single health care system and inability to classify acne severity or patient preference.

We found wide variation in the propensity to prescribe isotretinoin, even within a single health care system. A more rapid escalation of acne therapy is associated with a greater tendency to provide isotretinoin and may suggest changes in approaches to overall acne management to improve isotretinoin utilization.

## Conflicts of interest

None disclosed.
